# Fish consumption and risk of stroke: a second prospective case-control study from northern Sweden

**DOI:** 10.1186/s12937-016-0216-3

**Published:** 2016-11-16

**Authors:** Maria Wennberg, Jan-Håkan Jansson, Margareta Norberg, Staffan Skerfving, Ulf Strömberg, Per-Gunnar Wiklund, Ingvar A. Bergdahl

**Affiliations:** 1 Department of Public Health and Clinical Medicine, Nutritional Research, Umeå University, 901 87 Umeå, Sweden; 2 Department of Public Health and Clinical Medicine, Research Unit Skellefteå, Umeå University, Umeå, Sweden; 3 Department of Public Health and Clinical Medicine, Epidemiology and Global Health, Umeå University, Umeå, Sweden; 4 Division of Occupational and Environmental Medicine, Lund University, Lund, Sweden; 5 Health Metrics Unit, The Sahlgrenska Academy, University of Gothenburg, Gothenburg, Sweden; 6 Department of Public Health and Clinical Medicine, Medicine, Umeå University, Umeå, Sweden; 7 Department of Public Health and Clinical Medicine, Occupational and Environmental Medicine, Umeå University, Umeå, Sweden; 8 Department of Biobank Research, Umeå University, Umeå, Sweden

**Keywords:** Fish consumption, Ischaemic stroke, Hemorraghic stroke, Lifestyle, Confounding

## Abstract

**Background:**

Fish consumption has been concluded to be associated with decreased risk of stroke in several reviews. However, among men, but not women, an increased risk of stroke was previously found at high fish consumption (>3 meals/week) in northern Sweden. This study investigates if previous results on elevated stroke risk with high fish consumption in men in northern Sweden can be confirmed in a larger study with new cases in the same population.

**Methods:**

A prospective nested case-control study was performed within the population-based Northern Sweden Health and Disease Study cohort. Information on fish consumption, other lifestyle and medical data was collected at baseline. Incident stroke cases (1987–2007, *n* = 735) were identified and 2698 controls matched for gender, age, year of baseline and geographical region.

**Results:**

There were no associations between total fish or fatty fish consumption and stroke risk; thus the previous finding of increased risk of stroke with high fish consumption in men could not be repeated. High intake of lean fish (>twice/week compared to < once/month) was associated with increased stroke risk in men [OR 1.80 (95% CI 1.00, 3.21), but not in women [OR 0.50 (95% CI 0.24, 1.10)]. The association was driven by men living alone.

**Conclusions:**

The previous association between high total fish consumption and risk of stroke in men could not be repeated. The increased risk found in men with high intake of lean fish may be due to chance or confounding specific for this group.

**Electronic supplementary material:**

The online version of this article (doi:10.1186/s12937-016-0216-3) contains supplementary material, which is available to authorized users.

## Introduction

Fish consumption has been associated with protection against cardiovascular disease (CVD) in several epidemiological studies, reviewed by e.g. Wang et al. and Konig et al. [1, 2], and recently updated by Kromhout and de Goede [3]. Even though evidence is stronger for myocardial infarction, most reviews and meta-analyses indicate modest associations also between fish consumption and decreased risk of stroke [4–7]. However, we got conflicting results in our case-control study from northern Sweden [8], with an increased risk for stroke seen in men reporting fish consumption > 3 times/week as compared to those reporting fish consumption less than once a month, but no association for women. Lifestyle variables were not extensively adjusted for in that study; for example no information was presented for physical activity level or consumption of fruit and vegetables. A lifestyle study was therefore performed, examining associations between fish consumption and other health factors in over 60 000 men and women in the same region [9]. Fish consumption was associated with other healthy behaviors, such as non-smoking, high education, physical activity and higher consumption of fruit and vegetables, both in men and women. The only gender difference found was that high fish consumption was associated with consumption of all types of alcohol in men, but only with consumption of wine in women. The study could thus not explain the finding of increased risk of stroke in male high-consumers of fish found by Wennberg et al. [8], but underlined the importance of having control of other lifestyle variables in studies concerning fish consumption and health outcomes. We therefore wanted to repeat the study in a larger dataset.

This prospective study on fish consumption and stroke risk is based on a follow-up to year 2007 of the same cohorts that were studied by Wennberg et al. [8]. A larger questionnaire dataset facilitated a more extensive adjustment for lifestyle factors. The aim of the study was to find out if previous results on elevated stroke risk with high fish consumption in men in northern Sweden could be confirmed in a new study.

We believe that the previously observed association between high fish consumption and stroke risk in men, but not in women [8] could be explained by either of these alternatives (or a combination of these); i) it is a chance finding; ii): it is an effect of confounding differing between men and women; or iii): it is a true cause effect association.

## Methods

### Study population

The study population was derived from ongoing health examination programs for CVD and diabetes within the Northern Sweden Health and Disease study (NSHDS), which started in the mid 1980s; the Västerbotten Intervention Programme (VIP) [10, 11] and the WHO’s Multinational Monitoring of Trends and Determinants in Cardiovascular Disease (MONICA) study in northern Sweden [12, 13]. In the VIP, men and women living in the county Västerbotten are invited to participate in a health screening upon turning 40, 50 and 60 years (until 1996 also 30 years). In the MONICA Study, 2000 or 2500 randomly selected participants aged 25–74 years (the age group 65–74 years has been included since 1994) have been invited each screening year (the current study includes data from 1986, 1990, 1994, 1999 and 2004). The participation rates in the VIP have increased from approximately 55% in the early 1990s to around 65–70% from 2001, and have varied between 71.2 and 81.2% in the Northern Sweden MONICA study. By March 2007, 98,625 unique subjects had been screened in any of these two northern Sweden sub-cohorts, where data collection is similar. About 84% of the participants in the present study were from the VIP.

Participants were invited to answer a questionnaire on lifestyle variables, including a food frequency questionnaire (FFQ), and at the same occasion perform a medical examination. Health screening information was stored in a database.

### End points

#### Case identification

Cases were identified by screening for stroke in hospital medical records, primary care journals and death certificates according to the WHO MONICA criteria. All cases of transient ischemic attack, silent brain infarction (events without clinical signs), stroke caused by trauma, subarachnoid hemorrhage, and acute stroke with concomitant brain tumour or severe blood disease were excluded based on WHO criteria [12].

#### Case verification

In ischemic stroke no sign of hemorrhage on computerized tomography scan or autopsy was allowed, while hemorrhagic stroke was diagnosed through a positive finding on computerized tomography scan and/or autopsy. Unspecified strokes had neither tomography scan nor autopsy performed.

### Study design

**Fig. 1:**
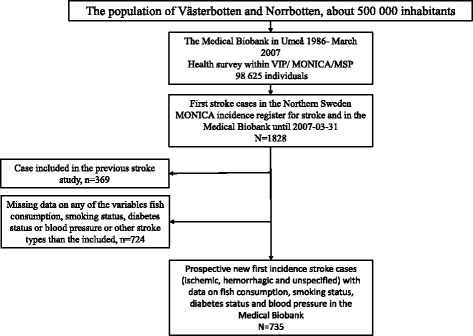
Identification of stroke cases and exclusions

To enable comparisons with our previous stroke study [8], a prospective nested case-control study design was used. By linkage between the health examination registry and the Northern Sweden MONICA Incidence Registry [12], those who previously participated in the VIP or the Northern Sweden MONICA Study and later on developed stroke could be identified. This study includes 735 stroke cases (627 ischemic strokes, 96 hemorrhagic strokes and 12 unspecified strokes) registered between September 1987 and March 2007 with 2698 controls (up to four controls per case) matched for age (±2 years), sex, date of health survey (±4 months), type of health survey (VIP or MONICA) and municipality. Only matched sets with information on fish consumption, smoking status, diabetes status and blood pressure for the case, and for at least one control were included in this study. Controls were excluded if they had died or moved out of the region before the event date of the matched case. Participants in the study were in the age span 29–74 years at baseline. In the previous northern Sweden stroke study [8] cases with previous stroke, myocardial infarction or cancer (according to the Swedish National Cancer Registry) were excluded. In this study those with previous stroke were excluded, but previous myocardial infarction or cancer were allowed. As a consequence, there are also cases in this study diagnosed before September 20, 2000, which was the latest date of stroke registry in the previous stroke study. Cases from our previous stroke study were not included in this study. Controls from the previous study may appear as case in this study, if they got a stroke after September 20, 2000 (Fig. 1).

### Baseline variables

Participants were categorized as non-smokers (including previous and occasional smokers) or current daily smokers from the questionnaire. Participants were considered as having diabetes if diabetes was self-reported in the questionnaire, if baseline fasting glucose was ≥7.0 mmol/L or if 2-h post-load plasma glucose was ≥11.0 mmol/L (≥12.2 mmol/L in the VIP, as capillary plasma was drawn). Systolic and diastolic blood pressures were measured twice at baseline and the mean value was recorded. Body mass index (BMI) was calculated as weight (kg)/height (m^2^). Total cholesterol was measured in serum, sampled after >4 h of fasting. Educational level was categorized to academic education (completed university degree) or not. Those reporting exercising in training clothes at least once a week or taking a walk at least twice a week was categorized as physically active.

In addition to these baseline variables we also considered civil status (living with other adult or not) in stratified analysis. The rationale behind this is that civil status is associated with cardiovascular health risks [14]. Missing data was not replaced.

### Dietary data

Dietary data was estimated from a FFQ, where average intake over the last year was asked for. There have been changes in the FFQ over time considering number of food items. The version currently in use in the Northern Sweden MONICA Study contains 84 food items. The same FFQ has been used in the VIP, but a shortened version of the FFQ, with 64 to 66 food items, was gradually implemented in the VIP from 1996. The FFQ has been validated against ten 24-h dietary recalls. Good reproducibility was demonstrated and a validity similar to that of FFQ measurements in other prospective cohort studies [15].

Questions concerning fish consumption were consistent in all FFQ versions. The questions asked about lean and fatty fish were: “How often do you eat lean fish (for example, perch, cod)?” and “How often do you eat fatty fish (for example, herring, lavaret, salmon)?” The pre-defined responses were quantified to meals per week as follows: never = 0, a few times a year = 0.05, 1-3 times per month = 0.5, once per week = 1.0, 2–3 times per week = 2.5, 4–6 times per week = 5.0, once per day = 7.0, 2–3 times per day = 17.5 and ≥ 4 times per day = 28.0. Consumption of lean and fatty fish was computed to total fish meals/week. Consumption of total fish was also consolidated into five categories; less than once per month, once per month to less than once per week, 1–2 meals per week, more than twice a week to three meals a week, and more than three meals per week. Consumption of lean and fatty fish was consolidated into four categories as for total fish, but the two highest categories merged. Estimated fish consumption from the FFQ has been validated against proportions of fatty acids from fish (EPA and DHA) in erythrocyte membranes and fair correlations were demonstrated (*R*_s_ = 0.42–0.51) [16].

Consumption of fruit and vegetables was dichotomized to at most once a day (sum of fruit and vegetables) or more than once a day. Frequencies of consumption of strong beer, wine and spirits were estimated from the FFQ.

### Dataset in comparison to the previous study

Similarities and differences between the present and previous study are presented in Table 1.

**Table 1:** Comparisons between the previous and present study on fish consumption and risk of stroke in Northern Sweden Health and Disease Study

	Previous study (Wennberg et al. 2007 [8])	Present study
Baseline	1986–1999	1986–2005
Number of cases/controls	369/738	735/2698
Cases diagnos year	1986–2000	1987–2007
Exclusions	any stroke, MI or cancer before baseline	any stroke before baseline, inclusion as case in the previous study
Fish consumption data	FFQ data; lean and fatty fish, blood concentrations of EPA, DHA and mercury	FFQ data; lean and fatty fish
Adjustment variables	Smoking, hypertension, serum-cholesterol, diabetes diagnosis, BMI, education	All in the previous study and alcohol consumption, consumption of fruit and vegetables, level of physical activity, civil status

### Statistical analysis

Calculations were performed separately for men and women. The Mann-Whitney t-test or the Chi square test was used to compare levels of baseline variables between cases and controls. Crude and adjusted odds ratios for stroke risk by fish consumption were tested with conditional logistic regression based on the matched case-control sets. The first multivariable model (Model 1) included established risk factors for CVD [17] that were associated with risk of stroke in this population; smoking, diabetes and systolic blood pressure. In model 2, other lifestyle or anthropometric baseline variables that differed statistically between all stroke cases and controls were added to model 1 (educational level, BMI, consumption of fruit- and vegetable and intake of wine). The same multivariate models were used for total stroke and stroke subtypes.

The statistical calculations were carried out using SPSS for windows (version 18.0–23.0; SPSS Inc., Chicago, IL, USA).

## Results

Baseline characteristics collected between 1986 and 2005 concerning 735 stroke cases (464 men and 271 women) and 2698 controls are presented in Table 2. Of the cases, 627 were diagnosed with ischemic stroke, 96 with hemorrhagic stroke and 12 could not be classified for stroke type. Mean fish consumption (lean fish + fatty fish) for the whole population was 1.18 meals/week (range 0–18.0 meals/week), of which 0.65 meals/week was lean fish (range 0–17.5 meals/week) and 0.53 meals/week was fatty fish (range 0–7.00 meals/week). For men, mean fish consumption was 1.13 meals/week (range 0–12.0 meals/week) and for women 1.25 meals/week (range 0–18.0 meals/week). Among stroke cases a larger proportion were smokers, had diabetes and hypertension compared to controls. Fewer stroke cases had academic education or high consumption of fruit- and vegetables (>once/day), compared to controls. Male stroke cases had higher BMI and lower intake of wine compared to controls (Table 2).

**Table 2:** Baseline characteristics for 735 stroke cases and 2698 controls in Northern Sweden Health and Disease study

	Controls		All stroke cases		Ischemic stroke		Hemorrhagic stroke	
	N	%/Mean (SD)/Median (25^th^, 75^th^ percentile)	N	%/Mean (SD)/Median (25^th^, 75^th^ percentile)	N	%/Mean (SD)/Median (25^th^, 75^th^ percentile)	N	%/Mean (SD)/Median (25^th^, 75^th^ percentile)
Smokers (%)	2698	25.7	735	36.2^a^	627	36.7^a^	96	30.2
Men	1691	27.4	464	36.4^a^	389	37.5^a^	66	25.8
Women	1007	22.7	271	35.8^a^	238	35.3^a^	30	40.0^a^
Academic education (%)	2669	14.5	725	11.6^a^	617	13.0	96	4.17^a^
Men	1677	12.7	461	9.54	386	10.9	66	3.03^a^
Women	992	17.5	264	15.2	231	16.4	30	6.67
Physically active (%)	2665	74.6	723	71.8	617	70.7	94	77.7
Men	1673	70.8	457	69.4	384	67.7	64	78.1
Women	992	80.9	266	75.9	233	75.5	30	76.7
>once/day Fruit- and vegetable (%)	2620	87.7	711	83.4^a^	607	83.2^a^	93	83.9
Men	1633	84.0	446	79.4^a^	375	78.4^a^	63	84.1
Women	987	93.9	265	90.2^a^	232	90.9	30	83.3^a^
Strong beer (intakes/week)	2622	0.05^b^ (0, 0.05)	708	0.05^b^ (0, 0.05)	605	0.05^b^ (0, 0.05)	93	0.05^b^ (0, 0.28)
Men	1642	0.50^b^ (0, 0.50)	451	0.50^b^ (0, 0.50)	378	0.50^b^ (0, 0.50)	65	0.05 (0, 0.50)
Women	980	0.00^b^ (0, 0.05)	257	0.00^b^ (0, 0.05)	227	0.00^b^ (0, 0.05)	28	0.00^b^ (0, 0.05)
Wine (intakes/week)	2654	0.05^b^ (0, 0.50)	712	0.05^a,b^ (0, 0.50)	607	0.05^a,b^ (0, 0.50)	94	0.05^b^ (0, 0.50)
Men	1661	0.05^b^ (0, 0.50)	449	0.05^a,b^ (0, 0.50)	376	0.05^a,b^ (0, 0.50)	65	0.05^b^ (0, 0.50)
Women	993	0.05^b^ (0, 0.50)	263	0.05^b^ (0, 0.50)	231	0.05^b^ (0, 0.50)	29	0.05^b^ (0, 0.50)
Spirits (intakes/week)	2659	0.05^b^ (0, 0.50)	718	0.05^b^ (0, 0.50)	615	0.05^b^ (0, 0.50)	92	0.05^b^ (0, 0.50)
Men	1670	0.05^b^ (0.05, 0.50)	456	0.05^b^ (0.05, 0.50)	384	0.05^b^ (0,05, 0.50)	64	0.50^b^ (0.01, 0.50)
Women	989	0.05^b^ (0, 0.05)	262	0.05^b^ (0, 0.05)	231	0.05^b^ (0, 0.05)	28	0.05^b^ (0, 0.05)
Age (years)	2698	55.3 (7.40)	735	55.4 (7.40)	627	55.4 (7.38)	96	55.0 (7.87)
Men	1691	55.4 (7.29)	464	55.5 (7.23)	389	55.5 (7.16)	66	54.9 (7.97)
Women	1007	55.3 (7.60)	271	55.2 (7.69)	238	55.2 (7.73)	30	55.0 (7.77)
Diabetes (%)	2698	5.37	735	11.7^a^	627	12.8^a^	96	6.25
Men	1691	5.14	464	12.1^a^	389	13.6^a^	66	4.54
Women	1007	5.76	271	11.1^a^	238	11.3^a^	30	10.0
BMI (kg/m^2^)	2689	25.8^b^ (23.6, 28.4)	734	26.4^a,b^ (24.2, 29.4)	626	26.2^a,b^ (24.1, 29.4)	96	26.8^a,b^ (24.5, 29.7)
Men	1684	25.9^b^ (24.0, 28.2)	464	26.8^a,b^ (24.7, 29.4)	389	26.7^a,b^ (24.7, 29.4)	66	27.1^a,b^ (24.6, 29.8)
Women	1005	25.4^b^ (23.0, 28.9)	270	25.7^b^ (23.1, 29.7)	237	25.6^b^ (22.7, 29.6)	30	25.8^b^ (24.0, 29.7)
Systolic BP (mmHg)	2698	135 (17.4)	735	142^a^ (19.0)	627	142^a^ (19.3)	96	144^a^ (17.5)
Men	1691	135 (16.9)	464	143^a^ (18.3)	389	143^a^ (18.7)	66	144^a^ (16.5)
Women	1007	134 (18.2)	271	141^a^ (20.1)	238	140^a^ (20.2)	30	144^a^ (20.0)
Serum cholesterol (mmol/L)	2681	6.11 (1.25)	731	6.08 (1.25)	624	6.09 (1.26)	95	6.06 (1.21)
Men	1679	6.03 (1.20)	462	5.98 (1.25)	388	6.00 (1.25)	65	5.93 (1.33)
Women	1002	6.25 (1.31)	269	6.25 (1.23)	236	6.25 (1.27)	30	6.34 (0.84)

^a^Statistically significant difference between cases and controls

^b^Levels are medians (25^th^, 75^th^ percentile)

In continuous analyses (intakes/week) there were no associations between consumption of total fish or fatty fish and risk of stroke. In men, consumption of lean fish was associated with an increased risk of stroke in the fully adjusted model [odds ratio (OR) 1.21 (95% confidence interval (CI) 1.02, 1.43) per intake/week] (Additional file 1A). When stratifying for stroke subtype no statistically significant associations were found, but for men the point estimate for ischemic stroke risk by lean fish consumption was similar as for all stroke risk [OR 1.18 (95% CI 0.95, 1.47)] (Additional file 1B and C).

**Fig. 2:**
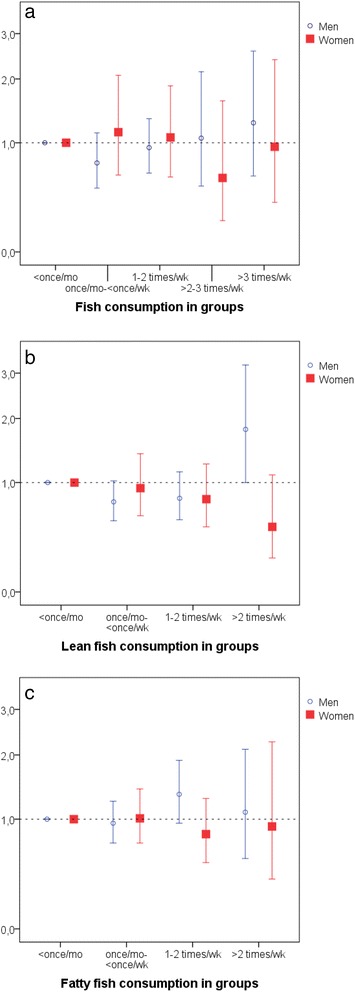
**a** Odds ratios of stroke risk in men and women by five categories of fish consumption, adjusted for diabetes status, smoking status, systolic blood pressure, BMI, educational level, consumption of fruit- and vegetables and consumption of wine. **b** Odds ratios of stroke risk in men and women by four categories of lean fish consumption, adjusted for diabetes status, smoking, status, systolic blood pressure, BMI, educational level, consumption of fruit- and vegetables and consumption of wine. **c** Odds ratios of stroke risk in men and women by four categories of fatty fish consumption, adjusted for diabetes status, smoking, status, systolic blood pressure, BMI, educational level, consumption of fruit- and vegetables and consumption of wine

Analyses with fish consumption in the same categories as in the previous study [8], showed no association for total fish consumption, in men or women (Additional file 2A, Fig. 2a). Separate analyses for lean and fat fish, using four intake categories (with the limit > 2 meals/week for the highest category because there were not sufficient individuals with higher consumption) and with full adjustment revealed a borderline statistically significant odds ratio of 1.80 (95% CI 1.00, 3.21) for men in the highest category of lean fish intake, compared to lean fish < once/month (Additional file 2B, Fig. 2b). The tendency of risk increase by lean fish for men was driven by the highest category of consumption (>2 meals/week of lean fish) (Fig. 2b). With moderate consumption of lean fish (once/month - 2 meals/week) a decreased risk of stroke was demonstrated in men, although not statistically significant in the fully adjusted model (Additional file 2B, Fig. 2b). No association with stroke risk was found for fat fish consumption in categories (Additional file 2C, Fig. 2c).

Men in the highest intake category of lean fish were to a higher degree living alone as compared to men in the other intake categories (25.3% as compared with 16.7% in the lower intake categories combined). Stratifying for civil status, men living alone (*n* = 35 complete sets providing data) had a fully adjusted OR for stroke of 3.49 (95% CI 0.97, 12.6) per intake/week increase of lean fish. The corresponding OR for men living together with someone (*n* = 324 complete sets) was 1.04 (95% CI 0.82, 1.32).

## Discussion

In this prospective study of 735 stroke cases with matched controls, the previous finding of a higher risk of stroke by high total fish consumption in men could not be repeated. For men, consumption of lean fish (per intakes/week) was associated with an increased risk of stroke in the fully adjusted model. Looking at fish consumption data in categories, there was a risk increase for men only in the highest consumption group of lean fish (>2 meals/week compared to < once/month), and this association was driven by men living alone.

The increased risk of stroke with high consumption of lean fish in men is interesting in light of our previous finding of increased risk of stroke in men with high total fish consumption (>3 meals/week) as compared to low consumption [8]. In our previous study, this was suspected to be a chance finding resulting from only few cases in the highest intake category (12 male cases and 15 male controls). Although this time only found for lean fish we again see an increased risk of stroke with high fish consumption in this larger study (26 male cases and 51 male controls in the highest intake category of lean fish) on the same population, and again only in men. In cohort studies from Western countries with sex specific analysis, inverse associations between fish consumption and risk of stroke are less convincing for men than for women [5, 18].

If there is really an increased risk of stroke with high consumption of lean fish in men, what could be the cause? Some predatory lean fish are high in methylmercury (pike and perch), which may cause cardiovascular damage at high concentrations [19, 20]. In our previous study we investigated associations between stroke risk and levels of mercury in erythrocytes and found no association [8]. Methylmercury was therefore not measured in the current study, and an increase in cardiovascular risk with methylmercury cannot be ruled out for those with the highest levels [20]. However, a decrease in body burden of mercury from 1990 until 1999 has been demonstrated in this population [21]. The decrease could not be explained by lower fish consumption only and therefore apparently reflects lower concentration of mercury in the fish consumed, either by a change to species lower in mercury and/or lower mercury concentrations in the locally caught fish. Taken together with the previous lack of association with methylmercury, we therefore do not believe that the finding on increased risk of stroke in men with high consumption of lean fish is due to methylmercury. There are other environmental pollutants in fish associated with cardiovascular risk, like persistent organic pollutants [22–24]. However, these are fat soluble and therefore found mainly in fatty fish, for which no association with stroke risk was found in this study.

We cannot rule out that high consumers of fish are to a larger extent risk patients of cardiovascular outcomes; fish is known to be a healthy food and risk patients may increase their consumption of fish because of that knowledge. However, then an association would rather be expected with fatty fish, more known of being beneficial.

A relatively high proportion of male high consumers of lean fish was living alone. When stratifying for civil status, it appeared that the risk increase by lean fish in men was driven by men living alone. Based on this observation, it can be speculated that confounding specific for this group may cause the association. For example, it is possible that the type of lean fish consumed differ between men living alone and men living in a relationship. More semi-finished products might be consumed by single men compared to men living in a relationship. In our study, we do not have information about cooking methods for the fish consumed. A common semi-finished lean fish product in Sweden is fish fingers, which is breaded, frozen fish that is fried in relatively large quantities of fat. As a consequence, single men with high lean fish consumption could be speculated to be large consumers of butter or margarine used for frying fish fingers. In the 80ties and early 90ties, margarines often contained trans fat which, as well as saturated fat in butter, has been found detrimental for cardiovascular health [25]. The hypothesis that intake of fried fish may increase risk of stroke is supported by previous studies finding an association between consumption of fried fish and risk of stroke or cardiovascular disease [26, 27].

The study has limitations. There is always a risk of misclassification in dietary surveys; people often do not remember correctly and also tend to overreport foods considered as healthy and underreport unhealthy foods. Fish consumption was estimated from a validated FFQ [15] designed to rank participants intake. Fair correlations were found between estimated intake of fatty acids from fish according to the FFQ and levels of the same fatty acids in erythrocytes [16], which is a strength. However, there is a risk that people participating in validation studies answers more carefully than people participating in general health examinations, with a more extensive questionnaire. There were some variations in the FFQ over time; however the fish consumption questions were consistent. Low variation in reported fish consumption may make any association difficult to detect; a majority of the participants (62%) belonged to the middle category reporting 1–2 meals/week of total fish consumption.

Strengths of the current study are the prospective design and the possibility to classify cases in stroke types; ischemic stroke and hemorrhagic stroke. Even though there were not enough cases to draw firm conclusions based on the analyses on hemorrhagic stroke, these could be separated from the ischemic strokes in the analyses and this did not reveal any major differences between ischemic and hemorrhagic stroke. A strength in this study was that cases and their controls answered the same FFQ, which makes differences in FFQs less likely to cause misclassification. Other strengths were that questionnaires were filled out before events occurred and cases and controls were matched for date of health survey, minimizing the risk of eg. changes over time in fish prices or pollutants in fish affecting the results.

It has been a concern that omega-3 fatty acids from fish would increase the risk of hemorrhagic stroke, because of the antiplatelet activity of these fatty acids [28]. In a recent meta-analysis of fish consumption and risk of stroke, a modest beneficial association was found between fish consumption and ischemic stroke, but no association was observed for hemorrhagic stroke [7]. In our study, no firm conclusions could be drawn on hemorrhagic stroke because of few cases, but the point estimate do not indicate that fish consumption would cause any risk of hemorrhagic stroke.

The recommendation from the Swedish National Food Agency to the general population is to eat 2–3 meals of fish per week, with a varied consumption of lean and fatty fish [29]. Even if lean fish consumption more than two meals per week would really increase the risk of stroke in men, the current recommendations can still be supported. The benefits of fish consumption are convincing concerning myocardial infarction [20], and the greatest health concern is that people do not eat enough fish.

## Conclusion

The gender difference found in our previous stroke study, with elevated risk of stroke for men with total fish consumption > 3 meals/week could not be repeated in this larger study from the same population. A risk increase was found in men with high consumption of lean fish, but this was limited to men living alone, suggesting that confounding specific for this group is the cause. Trans fatty acids from margarine, now no longer of concern, can be speculated to be involved. Future studies on fish consumption and risk of stroke should not be made without careful consideration of lifestyle factors associated with fish consumption as well as gender differences.

In conclusion, while yet an association between a fish consumption variable and stroke among men could be observed, we do not believe there is a cause effect association. In addition, the public health impact is small compared to the beneficial effects of fish. Therefore, the results support the current recommendations from the Swedish National Food Agency, of 2–3 meals of fish per week with a variation in species.

## Additional files

Additional file 1: A. Crude and adjusted odds ratio of all stroke by fish consumption in men and women. B. Crude and adjusted odds ratio of ischemic stroke by fish consumption in men and women. C. Crude and adjusted odds ratio of hemorrhagic stroke by fish consumption in men and women. (DOCX 27 kb)
Additional file 2: A. Crude and adjusted odds ratios for all stroke risk by categories of total fish consumption in men and women. B. Crude and adjusted odds ratios for all stroke risk by categories of lean fish consumption in men and women. C. Crude and adjusted odds ratios for all stroke risk by categories of fatty fish consumption in men and women. (DOC 92 kb)

## Abbreviations

BMI: Body mass index
CI: Confidence interval
CVD: Cardiovascular disease
FFQ: Food frequency questionnaire
NSHDS: Northern Sweden Health and Disease Study
OR: Odds ratio
The MONICA study: The Multinational Monitoring of Trends and Determinants in Cardiovascular Disease study
The VIP: The Västerbotten Intervention Programme
