# Supplementary material for: Fish consumption and risk of stroke: a second prospective case-control study from northern Sweden
Source: Nutr J. 2016 Nov 16;15:98. doi: 10.1186/s12937-016-0216-3 (PMC5112685; doi:10.1186/s12937-016-0216-3)
Supplement: Additional file 1: — A. Crude and adjusted odds ratio of all stroke by fish consumption in men and women. B. Crude and adjusted odds ratio of ischemic stroke by fish consumption in men and women. C. Crude and adjusted odds ratio of hemorrhagic stroke by fish consumption in men and women. (DOCX 27 kb) [file 12937_2016_216_MOESM1_ESM.docx]

Additional file 1A. Crude and adjusted odds ratio of all stroke by fish consumption in men and women

|  | N | Crude | | Model 1^a^ | | N | Model 2^b^ | |
| --- | --- | --- | --- | --- | --- | --- | --- | --- |
|  | cases/ ref | OR | (95 % CI) | OR | (95 % CI) | cases/ ref | OR | (95 % CI) |
| Total fish (intakes/week) |  |  |  |  |  |  |  |  |
| All | 712/ 2567 | 1.03 | (0.95, 1.12) | 1.02 | (0.93, 1.11) | 671/ 2319 | 1.01 | (0.91, 1.11) |
| Men | 451/ 1620 | 1.06 | (0.95, 1.18) | 1.06 | (0.94, 1.18) | 426/ 1457 | 1.10 | (0.97, 1.24) |
| Women | 261/ 947 | 0.99 | (0.87, 1.13) | 0.98 | (0.86, 1.12) | 245/ 862 | 0.89 | (0.75, 1.05) |
| Lean fish (intakes/week) |  |  |  |  |  |  |  |  |
| All | 725/ 2640 | 1.09 | (0.98, 1.21) | 1.09 | (0.98, 1.21) | 675/ 2346 | 1.06 | (0.93, 1.20) |
| Men | 459/ 1661 | 1.14 | (0.98, 1.33) | 1.16 | (0.99, 1.34) | 428/ 1472 | **1.21** | **(1.02, 1.43)** |
| Women | 266/ 979 | 1.04 | (0.89, 1.21) | 1.02 | (0.86, 1.20) | 247/ 874 | 0.83 | (0.65, 1.06) |
| Fatty fish (intakes/week) |  |  |  |  |  |  |  |  |
| All | 720/ 2613 | 0.98 | (0.86, 1.13) | 0.98 | (0.85, 1.13) | 678/ 2350 | 1.02 | (0.88, 1.18) |
| Men | 455/ 1644 | 1.03 | (0.88, 1.21) | 1.02 | (0.87, 1.21) | 429/ 1474 | 1.09 | (0.92, 1.29) |
| Women | 265/ 969 | 0.88 | (0.68, 1.14) | 0.89 | (0.68, 1.16) | 248/ 876 | 0.90 | (0.68, 1.19) |

^a^Model 1 = adjusted for traditional risk factors; smoking, diabetes status and systolic blood pressure

^b^Model 2 = Model 1 + additionally adjusted for consumption of fruit- and vegetable, intake of wine, educational level and BMI

Additional file 1B. Crude and adjusted odds ratio of ischemic stroke by fish consumption in men and women

|  | N | Crude | | Model 1^a^ | | N | Model 2^b^ | |
| --- | --- | --- | --- | --- | --- | --- | --- | --- |
|  | cases/ ref | OR | (95 % CI) | OR | (95 % CI) | cases/ ref | OR | (95 % CI) |
| Total fish (intakes/week) |  |  |  |  |  |  |  |  |
| All | 607/ 2194 | 1.03 | (0.94, 1.13) | 1.02 | (0.93, 1.12) | 573/ 1986 | 1.02 | (0.92, 1.13) |
| Men | 378/ 1360 | 1.06 | (0.94, 1.19) | 1.05 | (0.93, 1.18) | 358/ 1227 | 1.10 | (0.97, 1.25) |
| Women | 229/ 834 | 1.00 | (0.88, 1.15) | 0.99 | (0.86, 1.14) | 215/ 759 | 0.90 | (0.75, 1.08) |
| Lean fish (intakes/week) |  |  |  |  |  |  |  |  |
| All | 618/ 2254 | 1.07 | (0.95, 1.21) | 1.06 | (0.93, 1.21) | 574/ 2001 | 1.01 | (0.85, 1.19) |
| Men | 385/ 1394 | 1.08 | (0.89, 1.31) | 1.09 | (0.89, 1.34) | 359/ 1235 | 1.18 | (0.95, 1.47) |
| Women | 233/ 860 | 1.06 | (0.90, 1.25) | 1.04 | (0.88, 1.23) | 215/ 766 | 0.84 | (0.65, 1.10) |
| Fatty fish (intakes/week |  |  |  |  |  |  |  |  |
| All | 615/ 2237 | 0.98 | (0.85, 1.14) | 0.98 | (0.84, 1.14) | 579/ 2016 | 1.03 | (0.88, 1.20) |
| Men | 382/ 1382 | 1.04 | (0.87, 1.21) | 1.03 | (0.86, 1.22) | 361/ 1244 | 1.09 | (0.91, 1.31) |
| Women | 233/ 855 | 0.86 | (0.65, 1.15) | 0.89 | (0.66, 1.19) | 218/ 772 | 0.91 | (0.67, 1.23) |

^a^Model 1 = adjusted for traditional risk factors; smoking, diabetes status and systolic blood pressure

^b^Model 2 = Model 1 + additionally adjusted for consumption of fruit- and vegetable, intake of wine, educational level and BMI

Additional file 1C. Crude and adjusted odds ratio of hemorrhagic stroke by fish consumption in men and women

|  | N | Crude | | Model 1^a^ | | N | Model 2^b^ | |
| --- | --- | --- | --- | --- | --- | --- | --- | --- |
|  | cases/ ref | OR | (95 % CI) | OR | (95 % CI) | cases/ ref. | OR | (95 % CI) |
| Total fish (intakes/week) |  |  |  |  |  |  |  |  |
| All | 95/ 340 | 0.98 | (0.77, 1.25) | 0.96 | (0.75, 1.23) | 91/ 315 | 0.84 | (0.63, 1.11) |
| Men | 65/ 235 | 1.12 | (0.83, 1.50) | 1.12 | (0.82, 1.53) | 62/ 216 | 1.01 | (0.69, 1.45) |
| Women | 30/ 105 | 0.78 | (0.50, 1.22) | 0.74 | (0.46, 1.19) | 29/ 99 | 0.64 | (0.37, 1.10) |
| Lean fish (intakes/week) |  |  |  |  |  |  |  |  |
| All | 95/ 346 | 0.95 | (0.66, 1.37) | 0.91 | (0.62, 1.32) | 91/ 320 | 0.73 | (0.47, 1.15) |
| Men | 65/ 239 | 1.21 | (0.75, 1.96) | 1.13 | (0.69, 1.85) | 62/ 220 | 0.92 | (0.49, 1.68) |
| Women | 30/ 107 | 0.65 | (0.31, 1.33) | 0.56 | (0.24, 1.29) | 29/ 100 | 0.50 | (0.21, 1.19) |
| Fatty fish (intakes/week |  |  |  |  |  |  |  |  |
| All | 95/ 341 | 1.00 | (0.68, 1.49) | 0.99 | (0.65, 1.50) | 91/ 316 | 0.88 | (0.56, 1.38) |
| Men | 65/ 235 | 1.10 | (0.68, 1.76) | 1.21 | (0.72, 2.03) | 62/ 216 | 1.10 | (0.62, 1.93) |
| Women | 30/ 106 | 0.84 | (0.40, 1.73) | 0.72 | (0.33, 1.56) | 29/ 100 | 0.66 | (0.27, 1.58) |

^a^Model 1 = adjusted for traditional risk factors; smoking, diabetes status and systolic blood pressure

^b^Model 2 = Model 1 + additionally adjusted for consumption of fruit- and vegetable, intake of wine, educational level and BMI
