# Supplementary material for: Fish consumption and risk of stroke: a second prospective case-control study from northern Sweden
Source: Nutr J. 2016 Nov 16;15:98. doi: 10.1186/s12937-016-0216-3 (PMC5112685; doi:10.1186/s12937-016-0216-3)
Supplement: Additional file 2: — A. Crude and adjusted odds ratios for all stroke risk by categories of total fish consumption in men and women. B. Crude and adjusted odds ratios for all stroke risk by categories of lean fish consumption in men and women. C. Crude and adjusted odds ratios for all stroke risk by categories of fatty fish consumption in men and women. (DOC 92 kb) [file 12937_2016_216_MOESM2_ESM.doc]

Additional file 2A. Crude and adjusted odds ratios for all stroke risk by categories of total fish consumption in men and women

|  | N | Crude | | Model 1^a^ | | N | Model 2^b^ | |
| --- | --- | --- | --- | --- | --- | --- | --- | --- |
|  | cases/ ref | OR | (95 % CI) | OR | (95 % CI) | cases/ ref | OR | (95 % CI) |
| All |  |  |  |  |  |  |  |  |
| <once/month | 90/ 265 | 1.00 |  | 1.00 |  | 84/ 253 | 1.00 |  |
| once/month – <once/week | 153 /628 | **0.73** | **(0.54, 0.99)** | 0.78 | (0.57, 1.08) | 145/ 590 | 0.88 | (0.63, 1.22) |
| 1-2 intakes/week | 415/ 1561 | 0.79 | (0.60, 1.04) | 0.84 | (0.63, 1.12) | 394/ 1486 | 0.97 | (0.72, 1.30) |
| >twice/week – <3 intakes/week | 24/ 102 | 0.70 | (0.41, 1.17) | 0.73 | (0.42, 1.25) | 23/ 98 | 0.79 | (0.45, 1.39) |
| >3 intakes/week | 30/ 90 | 0.98 | (0.60, 1.61) | 0.96 | (0.57, 1.62) | 26/ 86 | 1.05 | (0.60, 1.82) |
| Men |  |  |  |  |  |  |  |  |
| <once/month | 68/ 186 | 1.00 |  | 1.00 |  | 63/ 176 | 1.00 |  |
| once/month – <once/week | 90/ 405 | **0.62** | **(0.43, 0.90)** | 0.68 | (0.46, 1.00) | 84/ 375 | 0.76 | (0.50, 1.13) |
| 1-2 intakes/week | 258/ 966 | 0.74 | (0.53, 1.01) | 0.79 | (0.56, 1.10) | 247/ 919 | 0.94 | (0.65, 1.33) |
| >twice/week – <3 intakes/week | 17/ 54 | 0.89 | (0.47, 1.67) | 0.87 | (0.45, 1.69) | 16/ 52 | 1.06 | (0.52, 2.14) |
| >3 intakes/week | 18/ 54 | 0.92 | (0.49, 1.72) | 1.00 | (0.51, 1.94) | 16/ 50 | 1.27 | (0.62, 2.58) |
| Women |  |  |  |  |  |  |  |  |
| <once/month | 22/ 79 | 1.00 |  | 1.00 |  | 21/ 77 | 1.00 |  |
| once/month – <once/week | 63/ 223 | 1.00 | (0.58, 1.74) | 1.02 | (0.58, 1.81) | 61/ 215 | 1.14 | (0.63, 2.07) |
| 1-2 intakes/week | 157/ 595 | 0.94 | (0.57, 1.56) | 0.99 | (0.58, 1.66) | 147/ 567 | 1.07 | (0.61, 1.87) |
| >twice/week – <3 intakes/week | 7/ 48 | 0.52 | (0.20, 1.31) | 0.57 | (0.22, 1.48) | 7/ 46 | 0.60 | (0.22, 1.61) |
| >3 intakes/week | 12/ 36 | 1.18 | (0.52, 2.64) | 1.00 | (0.42, 2.34) | 10/ 36 | 0.95 | (0.37, 2.39) |

^a^Model 1 = adjusted for traditional risk factors; smoking, diabetes status and systolic blood pressure

^b^Model 2 = Model 1 + additionally adjusted for consumption of fruit- and vegetable, intake of wine, educational level and BMI

Additional file 2B. Crude and adjusted odds ratios for all stroke risk by categories of lean fish consumption in men and women

|  | N | Crude | | Model 1^a^ | | N | Model 2^b^ | |
| --- | --- | --- | --- | --- | --- | --- | --- | --- |
|  | cases/ ref | OR | (95 % CI) | OR | (95 % CI) | cases/ ref | OR | (95 % CI) |
| All |  |  |  |  |  |  |  |  |
| <once/month | 191 /572 | 1.00 |  | 1.00 |  | 178/ 541 | 1.00 |  |
| once/month – <once/week | 308/ 1237 | **0.76** | **(0.61, 0.93)** | **0.76** | **(0.61, 0.95)** | 287/ 1173 | 0.81 | (0.64, 1.03) |
| 1-2 intakes/week | 185/ 743 | **0.74** | **(0.58, 0.94)** | **0.74** | **(0.57, 0.94)** | 173/ 704 | 0.80 | (0.61, 1.04) |
| >twice/week | 41/ 122 | 1.03 | (0.69, 1.54) | 1.03 | (0.67, 1.56) | 37/ 116 | 0.99 | (0.63, 1.54) |
| Men |  |  |  |  |  |  |  |  |
| <once/month | 137/ 397 | 1.00 |  | 1.00 |  | 127/ 368 | 1.00 |  |
| once/month – <once/week | 190/ 807 | **0.69** | **(0.53, 0.89)** | **0.72** | **(0.55, 0.94)** | 175/ 767 | 0.77 | (0.57, 1.02) |
| 1-2 intakes/week | 104/ 418 | **0.72** | **(0.53, 0.97)** | **0.71** | **(0.51, 0.97)** | 100/ 396 | 0.81 | (0.58, 1.14) |
| >twice/week | 28/ 55 | 1.50 | (0.89, 2.51) | 1.62 | (0.93, 2.79) | 26/ 51 | **1.80** | **(1.00, 3.21)** |
| Women |  |  |  |  |  |  |  |  |
| <once/month | 54/ 175 | 1.00 |  | 1.00 |  | 51/ 173 | 1.00 |  |
| once/month – <once/week | 118/ 430 | 0.90 | (0.62, 1.31) | 0.84 | (0.57, 1.24) | 112/ 406 | 0.93 | (0.62, 1.40) |
| 1-2 intakes/week | 81/ 325 | 0.80 | (0.53, 1.19) | 0.78 | (0.51, 1.17) | 73/ 308 | 0.80 | (0.51, 1.25) |
| >twice/week | 13/ 67 | 0.65 | (0.33, 1.29) | 0.57 | (0.28, 1.16) | 11/ 65 | 0.51 | (0.24, 1.10) |

^a^Model 1 = adjusted for traditional risk factors; smoking, diabetes status and systolic blood pressure

^b^Model 2 = Model 1 + additionally adjusted for consumption of fruit- and vegetable, intake of wine, educational level and BMI

Additional file 2C. Crude and adjusted odds ratios for all stroke risk by categories of fatty fish consumption in men and women

|  | N | Crude | | Model 1^a^ | | N | Model 2^b^ | |
| --- | --- | --- | --- | --- | --- | --- | --- | --- |
|  | cases/ ref | OR | (95 % CI) | OR | (95 % CI) | cases/ ref | OR | (95 % CI) |
| All |  |  |  |  |  |  |  |  |
| <once/month | 234/ 843 | 1.00 |  | 1.00 |  | 221/ 797 | 1.00 |  |
| once/month – <once/week | 336/ 1302 | 0.93 | (0.76, 1.13) | 0.93 | (0.76, 1.14) | 316/ 1235 | 0.97 | (0.79, 1.20) |
| 1-2 intakes/week | 125/ 419 | 1.04 | (0.81, 1.33) | 1.05 | (0.81, 1.36) | 118/ 398 | 1.10 | (0.84, 1.45) |
| >twice/week | 25/ 102 | 0.88 | (0.55, 1.42) | 0.87 | (0.53, 1.43) | 23/ 96 | 0.97 | (0.57, 1.65) |
| Men |  |  |  |  |  |  |  |  |
| <once/month | 145/ 523 | 1.00 |  | 1.00 |  | 136/ 492 | 1.00 |  |
| once/month – <once/week | 208/ 836 | 0.90 | (0.71, 1.16) | 0.88 | (0.68, 1.14) | 198/ 792 | 0.95 | (0.72, 1.24) |
| 1-2 intakes/week | 85/ 251 | 1.19 | (0.87, 1.63) | 1.23 | (0.88, 1.70) | 80/ 234 | 1.34 | (0.95, 1.90) |
| >twice/week | 17/ 67 | 0.94 | (0.52, 1.68) | 0.90 | (0.49, 1.65) | 15/ 61 | 1.09 | (0.56, 2.11) |
| Women |  |  |  |  |  |  |  |  |
| <once/month | 89/ 320 | 1.00 |  | 1.00 |  | 85/ 305 | 1.00 |  |
| once/month – <once/week | 128/ 466 | 0.97 | (0.71, 1.33) | 1.02 | (0.74, 1.41) | 118/ 443 | 1.01 | (0.72, 1.42) |
| 1-2 intakes/week | 40/ 168 | 0.82 | (0.54, 1.24) | 0.83 | (0.54, 1.26) | 38/ 164 | 0.82 | (0.52, 1.28) |
| >twice/week | 8/ 35 | 0.79 | (0.34, 1.79) | 0.87 | (0.36, 2.05) | 8/ 35 | 0.91 | (0.37, 2.26) |

^a^Model 1 = adjusted for traditional risk factors; smoking, diabetes status and systolic blood pressure

^b^Model 2 = Model 1 + additionally adjusted for consumption of fruit- and vegetable, intake of wine, educational level and BMI
